# Parental Smoking and Education as Determinants of Overweight in Israeli Children

**Published:** 2006-03-15

**Authors:** Michael Huerta, Haim Bibi, Jacob Haviv, Shimon Scharf, Michael Gdalevich

**Affiliations:** Ben-Gurion University of the Negev, Barzilai Medical Center Campus, District Health Office; Ben-Gurion University of the Negev, Barzilai Medical Center Campus, Department of Pediatrics, Ashkelon, Israel; Barzilai Medical Center Campus, District Health Office, Ashkelon, Israel; Barzilai Medical Center Campus, District Health Office, Ashkelon, Israel; Ben-Gurion University of the Negev, Barzilai Medical Center Campus, District Health Office, Ashkelon, Israel. Dr Gdalevich is also affiliated with Tel-Aviv University, Sackler Medical School, Department of Epidemiology, Tel-Aviv, Israel

## Abstract

**Introduction:**

Obesity is a well-recognized risk factor for many chronic diseases. Pediatric overweight is an especially severe problem because its childhood onset increases the overall length of exposure to the detrimental effects of overweight, accelerates the onset of chronic disease, and affects children's physical, psychological, and social development. Several parental traits have been shown to be associated with an increased risk for childhood overweight. In our study, we quantified the mutual effects of parental education and smoking on the risk of filial overweight in a large population-based sample of Israeli schoolchildren, adjusting for the effects of age, sex, and immigration status.

**Methods:**

Data were collected in 1997 and 2000 from 8623 Israeli schoolchildren aged 8 to 13 years in two cross-sectional samples. Overweight was defined as body mass index (BMI) of greater than the 85th percentile for age and sex, and severe overweight was defined as BMI greater than the 95th percentile for age and sex.

**Results:**

Mean BMI was positively associated with number of parental smokers for a child. Parental smoking was an independent risk factor for both overweight and severe overweight, with a dose–response relationship between the number of parental smokers and the risk of filial overweight. Children whose parents did not attend college were at increased risk for overweight (odds ratio [OR], 1.21; 95% confidence interval [CI], 1.03–1.42) and severe overweight (OR, 1.49; 95% CI, 1.09–2.05) compared with children whose parents both attended college. Children with one college-educated parent were at increased risk for severe overweight (OR, 1.31; 95% CI, 1.004–1.71) compared with children whose parents both attended college.

**Conclusion:**

Parental education and smoking are independent risk factors for filial overweight. Children of less-educated, smoking parents should be targeted for overweight prevention and intervention efforts. These findings should also be included as key messages in adult smoking prevention and cessation campaigns. Parents who smoke should be warned that not only is their own health at stake, but their children are also at increased risk for overweight and its associated diseases.

## Introduction

Obesity is a well-recognized risk factor for many chronic diseases, including hypertension, diabetes, and atherosclerotic heart disease (1). Pediatric overweight is an especially severe problem, because its childhood onset increases the overall length of exposure to the detrimental effects of overweight, accelerates the onset of chronic disease, and affects children's physical, psychological, and social development. The prevalence of pediatric overweight has increased over the last 3 decades (1,2), and its effects have reached global epidemic proportions (3). Effective planning and implementation of obesity prevention programs requires the recognition of risk factors for childhood obesity so that groups at increased risk can be targeted for intervention.

Several parental traits have been shown to be associated with an increased risk for childhood overweight (4-6). Parental smoking has been shown to be an independent risk factor for childhood obesity among Asian and European children (7,8). In addition, parental socioeconomic status (SES), often measured by proxy variables such as maternal or paternal education level, has been shown in several studies to exhibit an inverse relationship with childhood overweight, with a greater risk associated with lower SES (4,5,9). However, a systematic review of the literature has revealed that the relationship between SES in early life and childhood obesity has not been clearly defined (6). Furthermore, it has been suggested that some of the effects of parental education on childhood obesity may be a result of confounding rather than a true association (5,9). Immigration status may be a potential confounder of this association, because it is associated with childhood overweight (10) and parental education, partly due to the different social and cultural environments among native-born and foreign-born families.

The objectives of this study were to quantify the effects of parental education and smoking on the risk of filial overweight among a large population-based sample of Israeli schoolchildren while adjusting for the effects of age, sex, and immigration status.

## Methods

### Study population

Data were collected in two cross-sectional school-based health surveys conducted by the Barzilai Medical Center in the Ashkelon district of Israel during 1997 and 2000, with sampling methods used from Peled et al (11). All elementary schools in the district were included in the sampling frame and were eligible for selection. Schools were selected using a random cluster sample approach; 50% of eligible schools were included in the final selection, which included a representative sample of 9719 children ranging in age from 8 to 13 years.

### Data collection

Each child was weighed wearing shorts, a T-shirt, and no shoes. Scales were calibrated with standard weights before use. Barefoot standing height was measured by a stadiometer to a precision of ±0.5 cm. In addition, each child's parents filled out a questionnaire that included information on the child's sex, age, country of birth, date of immigration to Israel, and parental education level and smoking status (Table 1). Time since immigration was calculated as the number of years elapsed between immigration and completion of the study questionnaire. Additional survey items included the reporting of respiratory symptoms, allergies, dermatologic conditions, and performance on lung-function tests; however, these data were not analyzed within the context of our study. Study protocols were approved by the Israeli Ministry of Health ethical committee and the Southern District of the Israeli Ministry of Education.

### Definition of overweight and severe overweight

Body mass index (BMI) was calculated as weight/height^2^ (kg/m^2^). Current terminology for the definition of overweight in children is not completely standardized (12). Although the Centers for Disease Control and Prevention (CDC) classifies the childhood categories as *at risk of overweight* (85th to <95th percentile BMI for age and sex) and *overweight* (BMI ≥95th percentile), other groups, including the International Obesity Task Force, prefer the terminology used for adults and classify children as *overweight* or *obese *(12,13). In our study, we define *overweight* as BMI greater than the 85th percentile for age and sex, whereas *severe overweight*, a subset of the overweight category, is limited to BMI greater than the 95th percentile.

### Data analysis

Comparison of mean BMI values with demographic variables was carried out using *t *tests for independent samples and one-way analysis of variance. A linear regression model was used to test for an association between BMI value and time elapsed since immigration. Univariate and multivariate logistic regression analyses were used to quantify the effects of socioeconomic variables on the risk of overweight and severe overweight. In the logistic models, overweight and severe overweight — each categorized as a dichotomous variable — served as the dependent outcome variables. Independent categorical covariates included in the models were age, sex, immigration status (either Israeli born or immigrant from the Commonwealth of Independent States [CIS], i.e., countries of the former Soviet Union), time since immigration (categorized as fewer than 5 years or 5 years or more), number of parental smokers (zero, one, or two), and maternal and paternal education level (high school graduate or less vs any college or more). An additional composite variable was constructed to reflect combined parental education (neither parent attended college, one parent attended college, or both parents attended college). Finally, interaction terms between parental education and child's age and parental education and parental smoking were added to the multivariate models to assess the joint impact of the variables on the risk of filial overweight and severe overweight. A priori level of significance was defined as *P *≤.05. Statistical analysis was conducted using SPSS software (SPSS Inc, Chicago, Ill) and the PEPI suite of computer programs (14).

## Results

Data on age, sex, and immigration status were available for 8959 of 9719 children (92.2%) aged 8 to 13 years in 1997 and 2000. The vast majority (96.2%) were born either in Israel (77.1%) or the CIS (19.1%). The remaining 336 children (3.8%) were born in 35 different countries and were excluded from analysis because of their low individual frequencies. The final study sample included 8623 children. The sample size was sufficient to detect differences of 0.2 BMI units and odds ratios (ORs) of 1.2 or higher across immigration, education, and smoking categories at a power exceeding 80%.

Demographic characteristics of the study sample are shown in Table 1. Of the children studied, 90.1% were aged 8 to 11 years, and girls were sampled more often than boys (4725 vs 3898). One fifth of the children were born in the CIS, and almost half (48.9%) were exposed to at least one smoking parent. In slightly more than half of the families (51.5%), neither parent had received any formal education after high school.

Mean BMI values and 50th, 85th, and 95th percentile values, stratified by sex and age, are shown in Table 2. Mean, median (data not shown), and 85th percentile values (but not 95th percentile values) were consistently higher among girls than boys for all age groups.

Table 3 is a comparison of mean BMI values according to demographic variable categories. Mean BMI was slightly higher among children who were born in the CIS and immigrated to Israel than among children born in Israel. Mean BMI was positively associated with the number of smokers in the home, and a consistent inverse trend was demonstrated with paternal, maternal, and combined parental education levels, although this association was not statistically significant. No significant association was detected between categorized length of time since immigration and mean BMI value (*P* = .37). Linear regression analysis of BMI value by time since immigration as a continuous variable did not demonstrate a significant association (*P* = .69).

Table 4 shows the results of the univariate analysis of the risk of overweight and severe overweight. Immigrant status was not associated with an increased risk of either condition, whereas more parental smoking and a lower parental education level were found to be associated with a greater risk of both filial overweight and severe overweight.

Table 5 shows the results of multivariate logistic regression models for overweight and severe overweight. Combined parental education was considered a more appropriate predictor of the dependent variables than either paternal or maternal education because it was a better model fit and a more plausible explanation of the overall effects of education on children's health status. Thus, combined parental education was included in the final regression models. Parental smoking remained an independent risk factor for both overweight and severe overweight, with a dose–response relationship between the number of parental smokers and the risk of filial overweight. Children of families with the lowest level of combined parental education (neither parent attended college) were at higher risk for overweight (OR, 1.21; 95% confidence interval [CI], 1.03–1.42) and severe overweight (OR, 1.49; 95% CI, 1.09–2.05) than children whose parents both attended college. Children of families with an intermediate level of education (one parent attended college) were at increased risk for severe overweight (OR, 1.31; 95% CI, 1.004–1.71), whereas the estimate of increased risk for overweight in this group (OR, 1.19; 95% CI, 0.98–1.45) was not statistically significant. The addition of interaction terms between 1) parental education and child's age and 2) parental education and parental smoking did not affect the multivariate model results.

## Discussion

In this large, school-based population study of Israeli children, parental education and smoking were found to be independent risk factors for filial overweight and severe overweight. The effects of parental education on filial overweight are likely influenced by a complex array of social behaviors. Education is commonly regarded as being related to overall SES. In several large population-based studies of German schoolchildren, it was found that although SES could be defined by a broad range of variables, parental education was the variable most strongly related to childhood obesity (4,8). The adverse effects of lower SES on obesity are reported to increase with age (15); however, these effects are already present as young as age 6 years (4) and continue throughout childhood. In our study, lower individual paternal and maternal education levels were associated with an increased risk for overweight and severe overweight. However, the combined education level of both parents was a better predictor of risk than the individual level of either parent. Whereas children of families with less educated parents were at a significantly increased risk for overweight, this pattern was not found in families in which at least one of the parents received any formal education after high school. Perhaps the added knowledge, attitudes, or skills acquired and introduced into the home by even one parent, such as nutritional awareness, dietary habits, and a healthy lifestyle approach, may be enough to reduce a child's risk of overweight and obesity.

Several potential explanations for the association between low levels of parental education and childhood obesity exist (16). Parents with a higher educational level tend to breast-feed their children more than parents with a lower educational level, perhaps because they are informed about the advantages of breast-feeding (17). Differences in cultural and social norms between parents with higher and lower levels of education might be another reasonable explanation. Dieting and healthy weight-control practices, such as reduced high-energy and fat intakes and increased exercise, are more common among women of higher SES (18). In addition, children who attend schools of higher SES may have more negative attitudes toward obesity (19).

Additional findings of our study included an association between parental smoking and severe filial overweight and a dose–response relationship between parental smoking and the risk of overall filial overweight. These findings are supported by at least two similar studies in very different settings. In one large, population-based cross-sectional study of 5- to 7-year-old German children, parental smoking was found to be an independent risk factor for obesity among boys but not among girls (8). Furthermore, parents of obese children smoked more cigarettes per day than did parents of leaner children, even after adjustment for parental education level. In a study of 6- to 7-year-old children in Hong Kong, overweight was significantly associated with having a father who was a current smoker (7). The percentage of fathers who smoked increased with filial BMI, and smoking remained an independent risk factor even after ruling out potential confounding by paternal education, obesity, and SES. The finding that parental smoking was an independent risk factor for filial overweight in such diverse social and cultural settings as Germany, Hong Kong, and Israel supports the causality of this association (20), but the underlying mechanism responsible for the association is still unclear. Some authors have suggested that the relationship may be linked to childhood catch-up growth among children of low birth weight that was caused by smoking during pregnancy or to poor eating habits of smoking parents (7). It is also possible that parents engaging in one risky behavior such as smoking are more likely to engage in additional risk-related behaviors, such as poor household nutritional habits, which in turn makes their children more inclined to become overweight and obese. Additional research is needed to explore these potential mechanisms, including data collection on smoking duration and dietary habits of smoking and nonsmoking parents.

Two particular strengths of this study were the large number of children studied and the population-based origin of the study sample. These two factors make selection bias unlikely. However, because of the demographic composition of the district under study, Arab children were not represented. Israel is a mixed-culture society; it is important to study risk factors among Arab children as well because determinants of childhood overweight and obesity among Arab children may differ from determinants in the non-Arab population. Furthermore, we did not find a difference in the risk of overweight between native Israelis and immigrants from the CIS. This finding, combined with the finding of a slightly higher mean BMI among CIS immigrants, merits additional study; the interactions among various socioeconomic, behavioral, nutritional, demographic, and genetic factors in the shaping of a child's risk for overweight is beyond the scope of this work. Finally, although critical data such as sex and age were recorded by research staff members and height and weight were measured at the time of the study, other study variables such as parental education and smoking habits were collected by questionnaire, which introduces the potential for suboptimal validity. However, the questionnaire used in this survey was translated into Russian for the benefit of CIS immigrants and has been validated in similar school-based health studies conducted previously in the Ashkelon district (11).

The results of this study provide important information for health educators. We have identified the children of less-educated, smoking parents as a group to be targeted for overweight prevention and intervention efforts. However, this finding should also be included as a key message in smoking prevention and cessation campaigns aimed at adults. Parents who smoke should be warned that not only is their own health at stake but their children are also at increased risk for overweight, obesity, and their associated diseases.

## Figures and Tables

**Table 1:** Demographic Characteristics of Study Participants, Ashkelon District, Israel, 1997 and 2000 (N = 8623)

**Variable**	**Category**	**No. (%)**
Age, y	8-9	4287 (49.7)
	10-11	3481 (40.4)
	12-13	855 (9.9)
Sex	Boy	3898 (45.2)
	Girl	4725 (54.8)
Immigrant status	CIS^a^ immigrants	1706 (19.8)
	Native Israelis	6917 (80.2)
Parental smokers	Zero	4206 (51.1)
	One	2947 (35.8)
	Two	1078 (13.1)
Father's education	High school or less	4995 (64.4)
	Some college or more	2767 (35.6)
Mother's education	High school or less	4792 (57.7)
	Some college or more	3509 (42.3)
Combined parental education	Neither attended college	3953 (51.5)
	One attended college	1473 (19.2)
	Both attended college	2245 (29.3)

a Commonwealth of Independent States.

**Table 2:** Body Mass Index (BMI) of Study Participants, by Sex and Age, Ashkelon District, Israel, 1997 and 2000 (N = 8623)

**Sex**	**Age, y**	**Mean BMI (SD)**	**BMI **		

			**50th Percentile**	**85th Percentile**	**95th Percentile**
Male	8-9	16.23 (2.44)	15.71	18.36	21.25
	10-11	17.56 (2.93)	16.88	20.70	23.67
	12-13	17.94 (3.19)	17.12	20.93	24.72
Female	8-9	16.26 (2.49)	15.75	18.65	21.24
	10-11	18.06 (3.27)	17.36	21.46	24.61
	12-13	18.44 (3.24)	17.86	21.98	24.59

**Table 3:** Mean Body Mass Index (BMI), by Demographic Variables of Study Participants, Ashkelon District, Israel, 1997 and 2000 (N = 8623)

**Variable**	**Category**	**Mean (SD)**	***P* value**
Age, y	8-9	16.25 (2.47)	<.001
	10-11	17.84 (3.14)	
	12-13	18.16 (3.22)	
Sex	Boy	16.94 (2.83)	<.001
	Girl	17.19 (3.05)	
Immigrant status	CIS^a^ immigrant	17.22 (2.91)	.04
	Native Israeli	17.05 (2.96)	
Time since immigration	<5 years	17.13 (2.84)	.37
	≥5 years	17.27 (2.96)	
Parental smokers	None	16.86 (2.84)	<.001
	One	17.20 (3.03)	
	Two	17.46 (3.06)	
Father's education	High school or less	17.15 (3.01)	.05
	Some college or more	17.01 (2.82)	
Mother's education	High school or less	17.12 (3.01)	.26
	Some college or more	17.05 (2.85)	
Combined parental education	Neither attended college	17.15 (3.01)	.31
	One attended college	17.08 (3.01)	
	Both attended college	17.02 (2.78)	

a CIS indicates Commonwealth of Independent States.

**Table 4:** Univariate Analysis of Risk for Filial Overweight and Severe Overweight, by Parental Smoking Habits, Level of Education, and Child's Immigrant Status, Ashkelon District, Israel, 1997 and 2000 (N = 8623)

	**Overweight (BMI >85th Percentile)^a^**		**Severe Overweight (BMI >95th Percentile)^a^**	

	**OR (95% CI)^b^**	***P* Value**	**OR (95% CI)^b^**	***P* Value**

**Immigrant status**				

CIS^c^ immigrant	1.15 (0.97-1.34)	.11	.92 (0.71-1.18)	.50
Native Israeli	Reference		Reference	

**Parental smokers**				

None	Reference		Reference	
One	1.30 (1.13-1.49)	<.001	1.49 (1.19-1.86)	<.001
Two	1.54 (1.28-1.85)	<.001	1.48 (1.09-2.00)	.01

**Father's education**				

High school or less	1.23 (1.07-1.41)	<.001	1.30 (1.04-1.63)	<.001
Some college or more	Reference		Reference	

**Mother's education**				

High school or less	1.23 (1.08-1.40)	<.001	1.21 (0.99-1.50)	.06
Some college or more	Reference		Reference	

**Combined parental education**				

Neither attended college	1.32 (1.12-1.54)	<.001	1.49 (1.09-2.04)	.01
One attended college	1.23 (1.01-1.50)	.04	1.40 (1.08-1.82)	.01
Both attended college	Reference		Reference	

a BMI indicates body mass index.

b OR indicates odds ratio; CI, confidence interval.

c CIS indicates Commonwealth of Independent States.

**Table 5:** Multivariate Logistic Regression Analysis of Risk for Filial Overweight and Severe Overweight, by Parental Smoking Status and Education Level, Adjusted for Child's Age, Sex, and Immigrant Status, Ashkelon District, Israel, 1997 and 2000 (N = 8623)

	**Overweight (BMI >85th Percentile)^a^**		**Severe Overweight (BMI >95th Percentile)^a^**	

	**OR (95% CI)^b^**	***P* Value**	**OR (95% CI)^b^**	***P* Value**

**Parental smokers**				

Zero	Reference		Reference	
One	1.27 (1.10-1.47)	.001	1.47 (1.07-2.02)	.02
Two	1.47 (1.21-1.79)	<.001	1.49 (1.18-1.88)	<.001

**Combined parental education**				

Neither attended college	1.21 (1.03-1.42)	.02	1.49 (1.09-2.05)	.01
One attended college	1.19 (0.98-1.45)	.08	1.31 (1.004-1.71)	.049
Both attended college	Reference		Reference	

a BMI indicates body mass index.

b OR indicates odds ratio; CI, confidence interval.

c CIS indicates Commonwealth of Independent States.
